# Rectovaginal Fistulas, Their Surgical Management, and Outcomes: An Indian Experience

**DOI:** 10.7759/cureus.86400

**Published:** 2025-06-19

**Authors:** Ashok Kumar, Shahrukh Rauf, Manas Aggarwal, Yajnadatta Sarangi

**Affiliations:** 1 Surgical Gastroenterology, Sanjay Gandhi Postgraduate Institute of Medical Sciences, Lucknow, IND; 2 Gastrointestinal Surgery, Gastrointestinal Oncology, and Bariatric Surgery, Medanta Super Speciality Hospital, Lucknow, IND

**Keywords:** factors affecting surgical outcomes, martius flap, rectovaginal fistula, surgery for rectovaginal fistula, surgical repair

## Abstract

Background

Rectovaginal fistulas (RVFs) are abnormal epithelial-lined tracts between the rectum and vagina that significantly impact quality of life. Their etiology is varied, and most cases require surgical repair. Multiple surgical approaches exist, with varying success rates ranging from very low to high. However, data from India on surgical outcomes remain limited. This study, conducted at a tertiary care center in North India, aims to evaluate the outcomes of RVF repair and identify factors associated with surgical success.

Methodology

This was a retrospective analysis of prospectively maintained data of all patients (n = 72) with RVF who were managed between 2005 and 2023 at a tertiary care hospital in North India. IBM SPSS Statistics software for Windows, Version 26.0 (IBM Corp., Armonk, NY, USA) was used for statistical analyses. Continuous variables are presented as mean and standard deviation (SD) or median (interquartile range (IQR)), depending on normality status, while categorical variables are reported as raw values with percentages. Univariate analysis was performed using Pearson’s chi-square and Fisher’s exact tests. A p-value <0.05 was considered significant.

Results

A total of 72 patients were managed for RVF. Various etiological factors were iatrogenic injury in 23 (31.9%) patients, obstetric causes in 18 (25%) patients, malignancy in 19 (26%) patients, and congenital anomalies in five (7%) patients. Forty-eight (67%) patients required surgical intervention. Among those who underwent surgical intervention, various techniques were utilized: transvaginal layered closure in 24 (50%) patients, transvaginal advancement flap repair in 15 (31.3%) patients, transperineal layered closure with the Martius flap interposition in seven (14.6%) patients, and coloanal anastomosis in two (2.7%) patients. Fourteen (29%) patients required more than one surgical procedure. The overall success rate following surgical repair was 70.8%. No clinical or demographic factors were found to be statistically significant predictors of surgical failure.

Conclusions

In our study, the most common cause of RVF was iatrogenic injury. The majority of patients required surgical repair, with outcomes comparable to those reported in the literature. Among the various factors analyzed for their potential influence on surgical success, none were found to be significantly associated with the outcome.

## Introduction

Rectovaginal fistulas (RVFs) are abnormal epithelial-lined connections between the rectum and vagina. The incidence varies with etiology and demographics. In developing countries, obstetric fistulas are more common, while, in developed countries, fistulas are usually iatrogenic and often occur following rectal or vaginal surgeries. In a systematic review, the pooled prevalence in population-based studies was 0.29 (95% CI 0.00, 0.00,1.07) fistulas per 1,000 women of reproductive age across all regions, 1.60 (95% CI 1.16, 2.10) in Sub-Saharan Africa, and 1.20 (95% CI 0.10, 3.54) per 1000 in South Asia [1]. In a prospective Norwegian study, the incidence of obstetric fistulas was reported to be 16.3 per 100,000 deliveries, with RVFs being the most common (60%) [2]. The incidence of RVFs following anterior resection for rectal cancer ranges from 0.9% to 9.9% [3]. RVFs are classified according to their size and anatomical location [4,5]. It causes significant social distress and embarrassment and negatively impacts the patient's quality of life [6,7]. The initial aim of management should be infection control and improved hygiene, followed by definitive surgical management in the majority [8-10]. Several surgical options are available, depending on the patient's condition and the characteristics of the fistula [11]. Success rates following surgical repair vary widely, ranging from approximately 6% to 70% across different studies. Several factors have been associated with surgical failure [12-14]. There is a paucity of literature from India on the outcomes of surgical repair of RVFs. This study, conducted at a tertiary care center in North India, aimed to evaluate the surgical outcomes of RVF repair and to identify factors affecting the surgical success.

## Materials and methods

Study cohort

This was a retrospective analysis of a prospectively maintained database of all patients with RVFs over 18 years, from 2005 to 2023, in the Department of Surgical Gastroenterology at Sanjay Gandhi Postgraduate Institute of Medical Sciences, a tertiary care hospital in Lucknow, India. The study included all patients diagnosed with RVF, either clinically or via imaging. Information was retrieved from an electronically maintained hospital database. Preoperative clinical characteristics that could influence the outcome of surgical treatment were recorded for each patient, including demographic details, clinical presentation, etiology, and fistula characteristics. Follow-up data were collected through one-on-one interactions during outpatient visits or via telephonic conversation. All patients with RVFs diagnosed clinically or radiologically were included in the study. RVFs were classified according to their etiology, size, and location. They were categorized based on size as small (<0.5 cm), medium (0.5 cm to 2.5 cm), and large (>2.5 cm). According to their location in the vagina, they were classified as low type (located in the lower half of the vagina) or high type (situated in the upper half or the posterior vaginal fornix). 

Surgical technique

Depending on the type, location, previous repair failures, and local expertise, several surgical options are available and employed. Local repairs are utilized for small and simple fistulas, while advancement flaps are often used for refractory or larger fistulas. Complex fistulas necessitated transabdominal repairs, which ranged from sphincter-sacrificing bowel resection to permanent colostomy.

Failure of surgical repair was defined as the recurrence of symptoms following surgery. These patients subsequently underwent additional surgical repair. Several factors were analyzed to assess their impact on surgical outcomes, including comorbidities, etiology, obstetric history (primiparous vs. multiparous), symptom duration, previous repair attempts, presence of a diverting stoma, type of surgical repair, and fistula characteristics.

Statistical analysis

IBM SPSS Statistics software for Windows, version 26.0 (IBM Corp., Armonk, NY, USA) was used for statistical analyses. Continuous variables were presented as mean and standard deviation (SD) or as median and interquartile range (IQR), depending on data distribution, while categorical variables were reported as frequencies and percentages. Univariate analysis was performed using Pearson’s chi-square test and Fisher’s exact test. A p-value of <0.05 was considered statistically significant. Various risk factors for repair failure, such as etiology, fistula size, obstetric history, and duration of symptoms, were analyzed to identify factors associated with unsuccessful RVF repair.

## Results

This study included 72 patients, with a median age of 39 years (range 16-91 years). The median duration of symptoms was six months. Forty-six (63.8%) patients had symptoms for less than 12 months, while 26 (36.2%) patients had symptoms for more than 12 months. Sixty-seven (93.1%) patients presented with fecal and gaseous discharge per vaginum. The initial symptom was gaseous discharge only in the remaining five (6.9%) patients. Overall, 10 (13.8%) patients had a history of fecal incontinence, either for liquid or solid stools.

The etiological factors included iatrogenic injury in 23 (31.9%) patients, obstetric causes in 18 (25%) patients, malignancy in 19 (26.5%) patients, congenital anomalies in five (6.9%) patients, and trauma in two (2.8%) patients. The remaining five (6.9%) patients had disease-specific etiologies: Crohn’s disease in three, and tubercular and diverticular abscesses in one patient each. Twenty (27.8%) patients had a history of previous repair attempts. At presentation, 40 (55.5%) patients already had a diversion procedure, either a colostomy or an ileostomy. The majority of patients (53, 73.6%) had fistulas of sizes between 0.5 and 2.5 cm. The most common location of the fistula in relation to the vagina was the lower half, observed in 42 (58.3%) cases. The patient characteristics and the etiology of RVFs are detailed in Table 1, while fistula characteristics are presented in Table 2.

**Table 1:** Demographic features, clinical presentation, and etiology (n=72) COPD: chronic obstructive pulmonary disease; RHD: rheumatic heart disease

Clinical characteristics	Number of patients, n (%)
Age (median) (range)	39 (16-91 years)
Comorbidities	12 (16.6%)
Diabetes mellitus	05 (6.9%)
Hypertension	03 (4.2%)
Hypothyroidism	01 (1.3%)
COPD	02 (2.8%)
RHD	01 (1.3%)
Presenting symptoms	
Fecal and gaseous discharge per vaginum	67 (93.1%)
Only gaseous discharge per vaginum	05 (6.9%)
Preoperative disease duration (median)	
<12 months	46(63.8%)
>12 months	26(36.2%)
Etiology	
Iatrogenic	23 (31.9%)
Malignant	19 (26.5%)
Obstetric	18 (25%)
Congenital	05 (6.9%)
Miscellaneous (Crohn's related (3), diverticular abscess (1), tubercular (1))	05 (6.9%)
Traumatic	02 (2.8%)
Patients with repair attempted outside	20 (27.8%)
Previous diversion colostomy/Ileostomy	40 (55.5%)

**Table 2:** Clinical characteristics of rectovaginal fistulas (n=72)

Clinical characteristics	Number of patients, n (%)
Size of the fistula (cm)	
<0.5cm	10 (13.9%)
<0.5-2.5 cm	53 (73.6%)
>2.5 cm	09 (12.5%)
Distance of the fistula from anal verge	
<2 cm	28 (38.8%)
>2 cm	44 (61.1%)
Fistula in relation to vagina	
Lower half of vagina	42 (58.3%)
Upper half of vagina	30 (41.7%)
Fistula site in relation to the rectum	
Lower 1/3rd of the rectum	28 (38.8%)
Middle and upper 1/3rd of the rectum	44 (61.1%)

Management 

Twenty-four (33.3%) patients were managed conservatively with stool-bulking agents, a low-fiber diet, and maintenance of local hygiene. Of these, 17 (23.6%) patients had benign etiologies, low-lying fistulas, and minimal symptoms. All had a pre-existing diversion and experienced successful outcomes with conservative management. The remaining seven patients (9.7%) had fistulas secondary to malignant pathology and underwent various definitive treatments for the underlying malignancy. Forty-eight (66.6%) patients underwent surgical repair. Among them, 24 (50%) patients underwent transvaginal layered closure, 15 (31.3%) patients underwent transvaginal advancement flap repair, and seven (14.6%) patients underwent transperineal layered closure with the Martius flap interposition. A transabdominal approach was used in two (4.1%) patients. One of these had a complex RVF following radiotherapy for carcinoma of the cervix, with an associated rectosigmoid stricture. She underwent transabdominal resection of the strictured segment with colonic pull-through and coloanal anastomosis. The other patient was a case of iatrogenic high RVF involving the upper rectum and required resection of the diseased rectum, followed by colorectal anastomosis. The types of surgical procedures performed are summarized in Table 3.

**Table 3:** Types of surgical procedures performed (n=48)

Surgical procedure	n (%)
Transvaginal layered closure	24 (50%)
Transvaginal advancement flap	15 (31.3%)
Transperineal layered closure with the Martius flap	07 (14.6%)
Transabdominal approach	02 (4.1%)

Among the 48 patients who underwent surgical repair, 14 (29.2%) experienced symptom recurrence, which was classified as surgical failure. Analysis of potential factors influencing surgical outcomes showed no significant association with failure (Tables 4, 5).

**Table 4:** Analysis of the factors affecting the outcomes of surgical repair

Variable	Category	Successful (n=34) (70.8%)	Failure (n=14) (29.1%)	p-value
Etiology	Obstetric	09 (56.2%)	07 (43.8%)	0.178
	Traumatic	02 (100%)	0 (%)	0.891
	Iatrogenic	14 (87.5%)	02 (12.5%)	0.141
	Malignant	04 (80%)	01 (20%)	1.000
	Congenital	02 (50%)	02 (50%)	0.702
	Miscellaneous (Crohn’s disease, diverticular abscess, tubercular)	03 (60%)	02 (40%)	0.961
Presence of comorbidities	No	31 (72.1%)	12 (27.9%)	0.961
	Yes	03 (60%)	02 (40%)	
Obstetric history	None	26 (81.3%)	06 (18.8%)	0.056
	Primigravida	06 (60%)	04 (40%)	0.642
	Multigravida	02 (33.3%)	04 (66.7%)	0.093
Duration of disease	<12 months	22 (78.6%)	06 (21.4%)	0.283
	>12 months	12 (60%)	08 (40%)	
Earlier attempted repair	No	24 (70.6%)	10 (29.4%)	1.000
	Yes	10 (71.4%)	4 (28.6%)	
Diversion colostomy/Ileostomy	Present	15 (71.4%)	6 (28.6%)	1.000
	Absent	19(70.4%)	8 (29.6%)	
Type of surgical repair	Transvaginal layered closure	16 (66.7%)	8 (33.3%)	0.910
	Transvaginal advancement flap	11 (73.3%)	4 (26.7%)	0.752
	Transperineal layered closure with the Martius flap	5 (71.4%)	2 (28.6%)	1.020
	Transabdominal approach	2 (100%)	0	0.891

**Table 5:** Fistula characteristics and its impact on surgical outcome

Variable	Category	Successful 34(70.8%)	Unsuccessful 14(29.1%)	p-value
Location in the vagina	Lower half of the vagina	24 (70.5%)	10 (29.5%)	1.000
	Upper half of the vagina (fornix)	10 (71.4%)	4 (28.6%)	
Location with respect to the rectum	Lower 1/3^rd^ of the rectum	16 (72.7%)	6 (27.3%)	0.862
	Middle 1/3^rd^ or the upper part of the rectum	18 (69.2%)	8 (30.8%)	
Size of the fistula	<2.5 cm in diameter	30 (75%)	10 (25%)	0.151
	>2.5 cm	4 (50%)	4 (50%)	

Fourteen (29.1%) patients who experienced failure after the initial surgical repair underwent repeat surgical intervention (Table 6). The second repair was successful in all except one patient, despite multiple attempts. This patient initially presented with fecal incontinence secondary to an obstetric injury and underwent sphincteroplasty, levatorplasty, and vaginoplasty. Postoperatively, she developed an RVF and underwent multiple repairs, including the Martius flap, with failure, and at the time of completion of the study, she was being planned for coloanal anastomosis.

**Table 6:** Details of patients with failure of primary repair and repeat surgical repair Note: The numerical identifiers (e.g., 1, 2, 3, etc.) used in the table are arbitrary and were created solely for the purpose of referencing specific cases within this article. These identifiers do not correspond to any patient-identifying information.

Case No.	Age (in years)	Etiology	First repair	Second repair after failure	Final result
1	44	Iatrogenic	Transvaginal advancement flap	Transvaginal advancement flap	Successful
2	42	Obstetric	Transvaginal advancement flap	Transvaginal layered closure	Successful
3	34	Obstetric	Transvaginal advancement flap	Transvaginal advancement flap	Successful
4	51	Iatrogenic	Transvaginal advancement flap	Transvaginal layered repair, transvaginal layered repair, transperineal layered repair with the Martius flap, transperineal layered repair with the Martius flap	Failure
5	28	Obstetric	Transvaginal layered closure	Transvaginal layered closure	Successful
6	43	Obstetric	Transvaginal layered closure	Transvaginal repair with the Martius flap	Successful
7	46	Obstetric	Transvaginal layered closure	Transvaginal advancement flap	Successful
8	19	Congenital	Transvaginal layered closure	Transperineal repair	Successful
9	39	Obstetric	Transvaginal layered closure	Transvaginal layered closure	Successful
10	20	Obstetric	Transvaginal layered closure	Transperineal repair	Successful
11	53	Miscellaneous (Crohn’s disease)	Transvaginal layered closure	Transperitoneal repair with the Martius flap interposition	Successful
12	26	Congenital	Transvaginal layered closure	Transvaginal layered closure	Successful
13	32	Miscellaneous (Crohn’s disease)	Transperineal layered closure with the Martius flap	Repeat Martius flap interposition	successful
14	21	Malignant	Transperineal layered closure with the Martius flap	Transvaginal repair with mucosal advancement flap	Successful

## Discussion

RVF remains a formidable surgical challenge due to its complex anatomy, variable etiology, and high risk of recurrence. Although spontaneous healing may occur in some select cases, most require surgical intervention, with recurrence rates reaching up to 30%, irrespective of the repair technique employed [12]. Obstetric injury is the most commonly reported cause in the literature (up to 88%) [9,13]. However, in our series, iatrogenic injury was the leading cause in 23 (31.9%), followed by obstetric injury in 18 (25%) patients, likely reflecting referral bias as our hospital is a tertiary care referral center. Inflammatory bowel disease (IBD) has been reported in 2% to 23% of cases [15]. The 4.1% incidence of Crohn’s disease in our series was not different from the published series.

The initial goal of management should be the control of sepsis, including prompt treatment of any active infection. A diverting stoma is often one of the most important initial steps in managing rectovaginal fistula, as it helps control sepsis and alleviate the patient's symptoms [16,17]. Conservative treatment has led to fistula healing in 17 (23.6%) patients, as reported in various studies [18,19]. Conservative management is most effective for low-lying and small fistulae (less than 0.5 cm in diameter). Oakley et al. [19] reported that almost half of conservatively treated RVFs were expectantly small (<0.5 cm). In our study, all patients who were managed conservatively had low-lying fistulae.

Surgical intervention was required in 48 (66.6%) of our patients. Factors such as etiology, size, and location of the fistula, the condition of surrounding tissue, comorbidities, and prior repair attempts were used to determine the type of surgical approach [14]. The commonly performed local procedures for RVF are layered closure, rectal advancement flaps, and vaginal advancement flaps. While the transrectal approach allows access from the high-pressure side and facilitates easier mucosal mobilization, the transvaginal approach offers the advantage of better vascularized tissue and may promote faster recovery [15]. In our study, transvaginal layered closure was the most commonly performed procedure, with successful healing achieved in 16 out of 24 patients (66.7%), aligning with previously reported outcomes ranging from 50% to 100% [20,21]. This technique is ideal for small, low-lying, and uncomplicated RVFs, especially those of obstetric origin. Transvaginal advancement flap repairs were performed in 15 (31.3%) patients, particularly in cases with associated sphincter defects. This approach achieved successful healing in 11 out of 15 cases (73.3%), consistent with outcomes reported in the published literature [20,21]. 

Transvaginal advancement flap repair was employed in 15 (31.3%) patients, offering the added benefit of concomitant sphincteroplasty. This technique was preferred for patients with associated sphincter injury and incontinence. This approach achieved successful healing in 11 out of 15 cases (73.3%), consistent with outcomes reported in the published literature [20,21]. 

The Martius flap, using a well-vascularized labial fat or bulbocavernosus muscle pedicle, is employed for complex or recurrent fistulas to reinforce the vaginal and rectal walls. Reported success rates in literature range from 65% to 100%, with lower rates (65% to 70%) in radiation-induced or Crohn's-related fistulas [22]. The Martius flap provides robust vascular support and is particularly useful in cases with compromised tissue from previous radiation or inflammation [22]. In our study, a transperineal approach with Martius flap interposition was employed for complex and recurrent fistulas, achieving a healing rate of 71.4% (five out of seven cases). Transabdominal repair with or without diverting colostomy is particularly used for high, recurrent, and complex rectovaginal fistulas, including those secondary to inflammatory bowel disease, pelvic malignancy, and radiation [23]. In our study, transabdominal repairs were reserved for complex or high RVFs, particularly those associated with malignancy or radiation. Both patients in this group had successful outcomes. This approach is supported by Maggiori et al. [24] and others, who report success rates of 79% to 85% with techniques like colonic pull-through or coloanal anastomosis [25].

The overall success rates of RVF repair reported in various studies range from 46% to 88% (Table 7). This wide variability may be attributed to differences in patient selection, surgical techniques, and the level of surgical expertise. In our series, the overall success rate of 70.8% was similar to that reported in previously published series (Table 7).

**Table 7:** Published series on the surgical repair of rectovaginal fistulas and their outcomes

Serial No.	Study	Sample size	Surgical procedure	Success rate (%)
1.	Pinto et al.,2010 [26]	125	Advancement flap	88.2%
			Sphincteroplasty	
			Gracilis transposition	
			Martius flap	
			Seton, direct suture, plug, fibrin	
2.	Byrnes et al., 2017 [27]	107	Local Seton or plug, or glue (32)	46.4%
			Transvaginal or rectal (27)	
			Diverting stoma alone (14)	
			Transabdominal (34)	
3.	Fu et al., 2019 [28]	63	Transanal fistulectomy and suture of the rectum and vagina (35)	71.4%
			Transanal advanced flap (28)	
			Martius flap (10)	
			Gracilis interposition (3)	
			Transperineal approach (3)	
			Transvaginal approach (1)	
4.	Ryoo et al., 2019 [29]	92	Laparotomy (1)	42.4%
			Local repair [trans perineal (26), transvaginal (14]	
			Endorectal (1)	
			Local repair [trans perineal (26) transvaginal (14)	
5.	Studniarek et al.,2021 [30]	124	Advancement flap procedures (22)	73.4%
			Abdominal resections (16)	
			Primary closure with sphincter repair (17)	
			Fistula plug and fibrin glue (4)	
6.	Abo-Alhassan et al., 2022 [31]	100	Advancement flap (45)	60.3%
			Primary closure (40)	
			Ovesco clip ( 27)	
			Plug (24) and fibrin glue (19)	
			Rectal resection with colorectal or coloanal anastomosis (34)	
			Closure with sphincterorraphy (10)	
			Fistulotomy (7)	
			Rerouting Seton (3)	
			Martius flap (5), Gracilis flap (1)	
			Primary closure (40)	
7.	Present study	48	Transvaginal layered closure (24)	70.8%
			Transvaginal advancement flap (15)	
			Transperineal layered closure with a Martius flap (7)	
			Transabdominal approach (2)	

Obstetric RVFs generally have a higher likelihood of successful closure, often attributed to the younger age and better tissue quality of affected patients. In contrast, non-obstetric RVFs such as those associated with inflammatory bowel disease or radiation are challenging to treat due to ongoing inflammation, tissue fibrosis, or compromised vascularity [32]. In the present study, various factors were analyzed to see their effects on the surgical outcome, but none were found to be statistically significant. This could be due to the small number of patients in each group. 

The most common comorbidities in women with RVF include obesity, hypertension, and diabetes. In a study, Frontali et al. [32] found that comorbidities had no significant impact on RVF repair failure, consistent with our findings. However, it is widely accepted that optimizing comorbid conditions such as diabetes and hypertension can help reduce systemic inflammation and may enhance fistula healing. The protective role of diversion stoma in RVF prognosis remains unproven; however, it is often the first step to manage symptoms and achieve disease control [18].

Barugola et al. [33], in their study, showed the positive effect of diversion stoma on the healing of RVF. A contrasting study by Lambertz et al. [34] reported that the presence of a diversion stoma does not affect RVF recurrence. Pinto et al. [26] reported a 95% fistula recurrence rate in smokers, while non-smokers had significantly higher closure rates (43.8%) compared to current or former smokers (22.1%). Similarly, Manne et al. [12] identified smoking as a predictor of recurrence after fistula repair. They reported that fistula size primarily influences the surgical approach; small, low-type fistulas are often amenable to local repair, while larger, high-type, or recurrent fistulas typically require a transabdominal approach. Similarly, in our study, 17 (23.6%) patients experienced spontaneous resolution with a diverting stoma.

Our study has several limitations. First, its retrospective design may introduce bias and misclassification, as much of the follow-up data was record-based. Second, the sample size was limited, reducing statistical power. Despite these limitations, to the best of our knowledge, this is one of the largest single-institution studies from India to date, detailing various repair strategies, their outcomes, and factors influencing RVF repair [35, 36].

## Conclusions

RVFs have diverse etiologies, with iatrogenic causes being the most common in our study, followed by obstetric injuries. These fistulas are challenging to manage and often require surgical intervention using various approaches. Despite the complexity of management, surgical repair achieved a favorable overall success rate. In our study, no specific clinical or etiological factors were identified as having a significant impact on surgical outcomes.
